# Body image and willingness to change it—A study of university students in Poland

**DOI:** 10.1371/journal.pone.0293617

**Published:** 2023-11-01

**Authors:** Anna M. Platta, Anna T. Mikulec, Monika Radzymińska, Millena Ruszkowska, Grzegorz Suwała, Marek Zborowski, Przemysław Łukasz Kowalczewski, Marcin Nowicki

**Affiliations:** 1 Faculty of Management and Quality Science, Gdynia Maritime University, Gdynia, Poland; 2 Faculty of Engineering Sciences, University of Applied Science in Nowy Sącz, Nowy Sącz, Poland; 3 Faculty of Economic Sciences, Institute of Management Science and Quality, University of Warmia and Mazury in Olsztyn, Olsztyn, Poland; 4 Department of Food Product Quality, Krakow University of Economics, Kraków, Poland; 5 Faculty of Health Sciences, University of Applied Science in Nowy Sącz, Nowy Sacz, Poland; 6 Department of Food Technology of Plant Origin, Poznań University of Life Sciences, Poznań, Poland; 7 Department of Entomology and Plant Pathology, University of Tennessee, Knoxville TN, United States of America; Szkoła Główna Handlowa Warsaw School of Economics, POLAND

## Abstract

The present study aimed to identify declared patterns of ideal appearance among students at selected higher education institutions in Poland. In the present study, we set out to identify the body image and the propensity to change it. In order to achieve the goal of the study, a nationwide survey was carried out using a voluntary diagnostic survey. A total of 810 respondents took part in the survey. The study was conducted using a custom-made self-designed survey questionnaire with an indirect interview technique via a web-based platform (CAWI). The study results indicate that women were far more eager to maintain an attractive appearance through dieting than men. Importantly, the respondents, both women and men, did not identify influencers as role models of attractive appearance. Results of the present survey reveal that attitudes towards one’s physical appearance are diverse and influenced by the considered factors. The attributes that determined the perception of oneself as an attractive person included height, weight, and body build. Normal body weight is a key feature of an attractive appearance, according to the respondents; nevertheless, students show a higher tolerance for being overweight than for being underweight. Furthermore, preferences in this respect are largely conditioned by individual personal characteristics. According to young people, a person with an attractive appearance is one who eats and exercises regularly, and who does not fall into extremes in terms of their appearance. On the other hand, the fear of growing fat and losing one’s attractive appearance was frequently indicated among the concerns over personal appearance. Only one in five respondents expressed complete satisfaction with how they looked, with men and those with a normal BMI significantly more likely to do so. Respondents expressed readiness to undertake a range of activities related to increased exercise regime, changes in their eating habits, or seeking assistance (e.g., of a personal trainer) to achieve the desired body shape. Our findings can be used as a basis for discussion and consideration in developing educational activities on nutrition, as well as on overweight- and obesity-related health issues.

## 1. Introduction

Rapid progress of civilization has brought changes to the lifestyles of both young people and adults [1]. This extends to the day-to-day behaviours and functions of individuals at work and at home. According to the WHO, 60 per cent of factors related to an individual’s health and quality of life are correlated with lifestyle [2].

The WHO defines health as “a state of complete physical, social and mental well-being and not merely the absence of disease or infirmity.” “Physical” and “mental” well-being in this definition is closely related to the body and its perception [3]. Overweight and obesity are on the rise in both developed and developing countries, posing a huge challenge for health care [4]. In turn, excessive activity associated with the thin ideal promulgated by the media can lead to altered perceptions of body image, resulting in dissatisfaction with one’s own appearance and exaggerated over about one’s current weight. The definition of body image is perceived as the perception, thoughts and emotions accompanying the reaction to the perception of one’s own body. Moreover, this term is related to the mirror image of the body as a reflection of a social aspect conditioned by the culture and norms of a given society. This concept is created using body ideals, generally communicated through the media, family and peers [5]. Body image disorders are quite common in the general population. Reduced food intake correlated with weight manipulation, in combination with other environmental factors, may increase the risk of developing eating disorders [6]. According to the ICD-11 classification [7] feeding and eating disorders include: Anorexia Nervosa [8, 9] Bulimia Nervosa [9, 10], Binge Eating Disorder [11, 12], Avoidant-Restrictive Food Intake Disorder [13, 14], Pica [15, 16], Rumination-Regurgitation Disorder [17, 18] and other Specified Feeding or Eating Disorders [7]. Negative body image and appearance anxiety are associated with the risk of body dysmorphia [19, 20]. The body dysmorphic disorder is a disorder characterized by disturbing or imagined perceptions of one’s body and defects in physical appearance. Research conducted among the adult population in the United States indicates that 2.4% (49/2048) may suffer from dysmorphic disorders (by gender: 2.5% for women, 2.2% for men [21, 22]. A systematic review conducted in 2016 found that the estimated prevalence of the dysmorphic disorder in the general population was approximately 1.9–2.2% [23, 24]. Although men with muscular dysmorphia are often admired and gain public approval for their self-control in adhering to training plans and dietary practices, this disorder is correlated with reduced quality of life [25]. Dissatisfaction with one’s own appearance, adoption of strictly-controlled intensive workouts and restrictive diets, as well as avoidance of social contact and disruption of interpersonal relationships are the symptoms of another eating disorder, namely bigorexia nervosa. This disorder afflicts mainly men [26], but it has also been increasingly observed in young women [3]. Physical dysmorphia is synonymous with “reverse anorexia”, which was first described in the literature in the 1980s on the example of a group of body-builders [27]. Bigorexia encompasses environmental factors, such as social media influence, lack of self-confidence, peer pressure and eating attitudes, as well as personality traits, such as perfectionism [3]. Eating disorders (anorexia nervosa, bulimia nervosa, binge eating disorder, and other eating disorders) affect young people around the world. A narrative review by Silen [28] on the prevalence of eating disorders according to the Diagnostic and Statistical Manual of Mental Disorders (DSM-5) among young people from 2013 to 2022 indicates that a total of 5.5–17.9% of young women and 0.6–2.4% of young men experienced DSM-5 eating disorders in early adulthood.

The scientific achievements regarding body image and research showing its relationship with mental and physical health problems and behaviors are very extensive [29]. A significant proportion of research in this field, which can be found in literature, has been dedicated to women. Gagne et al. [30] assessed the aspects of body satisfaction in a sample of over 1,800 women aged 50 and older. About 62% of the respondents confirmed that their weight/shape negatively affected their lives, at least from time to time. Women with higher BMIs expressed greater concerns over weight and shape, whereas those with lower BMIs reported increased skin dissatisfaction. Additionally, it was found [31] that BMI predicted body dissatisfaction, body shape concerns, and striving for thinness only in young adult women (*age M* = 27.5 years), while it did not predict body dissatisfaction in older adult women (*age M* = 72.9 years). In contrast, a study [32] conducted with women aged 42–52 years found that those who were classified as body dissatisfied were twice as likely to report clinically significant symptoms of depression, compared to the women who were satisfied with their bodies. In addition, it has been demonstrated [33] that perimenopausal women reported significantly higher body image anxiety. Social comparison, appearance investment, and anxiety about aging are thought to mediate the negative effects of media exposure on body image in adult women aged 35 to 55 [34]. Research also suggests that when younger adult women (aged 18–28) view media images, social comparisons may mediate the relationship between image type and increased body dissatisfaction [35]. There are many reasons to suspect that adult women’s body image may differ and be more complex than in younger women [29].

Despite the abundance of body image research, few studies only have directly compared body image perceptions between women and men. As reported by Brenana et al. [36], body dissatisfaction in the group of young adults was more common and felt more strongly in women; however, men were also clearly affected in this respect. Another study [37] has also shown that women in late adolescence are more dissatisfied with their bodies compared to men. This tendency was also observed in children [38] and adolescents [39]. Ample studies have also shown that there is both an ideal male body identified with a muscular and well-shaped figure, and an ideal female figure perceived as slim and slender [30, 37, 40].

Considering the extensive literature addressing body dissatisfaction, the research on body image disorders in young adults remains an underrepresented domain. Research devoted to this issue should be considered relatively fragmentary and not exhaustive. Moreover, young people represent an interesting segment for studying health attitudes and behaviors due to their high consumer autonomy and purchasing power [41, 42] strong household influence [43] and insight into adults’ behavior [44]. Taking into account young respondents, university students were selected as a survey group in the present study. The university life and the resulting change in the social environment is a special time in terms of changes in the level of physical activity and eating habits, which may increase the risk of obesity [45–47]. The process of studying at the university is also a complex experience for young people, during which they encounter factors that predispose them to increased susceptibility to mental disorders [48]. Anxiety connected with own appearance is the experience of being judged by others based on physical appearance, which may negatively impact individuals in their social life, including their academic and professional life [3]. In addition, university students represent a group at high risk of developing eating disorders (ED) [49, 50]. Due to changing trends as well as the increasing role, importance and pressure of social media, it becomes important to monitor health attitudes and behaviors among young people. Therefore, in this study, we decided to identify body image and the propensity to change it among students of selected universities in Poland. The issues discussed in the article can be considered important both from the cognitive/scientific point of view but also from the standpoint of prevention and psychoeducation. This justifies the purposefulness of the study presented in this manuscript.

## 2. Research methodology

### 2.1. Subjects

In order to achieve the goal of the study, a nationwide survey was carried out using a voluntary diagnostic survey. A total of 810 respondents participated in the survey, including 461 women (representing 56.9% of the surveyed population) and 344 men (representing 42.5% of the surveyed population). Five people or 0.6% of the study population declined to state their gender. The gender of the respondents proved to be a differentiating factor in the surveyed population in terms of the perception of female and male image patterns, as well as the evaluation of one’s own appearance in the context of striving for individually assumed patterns.

The study group consisted of students from several universities at various levels of education, representing the following educational paths: first-cycle (undergraduate) students accounted for 90.4% of the study sample (732 people), second-cycle (graduate) students accounted for 9% of the study sample (73 people), and students of combined bachelors/masters programs accounted for 0.6% (5 people). Participants declared one of 14 voivodeships (provinces) of origin: Pomorskie (59.1% of respondents), Małopolskie (11.2% of respondents), Warmińsko-Mazurskie (9.3% of respondents), Kujawsko-Pomorskie (4.6% of respondents), Podlaskie (4.0% of respondents) Mazowieckie (2.3% of respondents), Zachodniopomorskie (2.0% of respondents), Lubelskie (1.9% of respondents), Podkarpackie (1.6% of respondents), Śląskie (1.2% of respondents), Łódzkie (1.0% of respondents), Wielkopolskie (0.9% of respondents), Świętokrzyskie (0.7% of respondents), and Dolnośląskie (0.2% of respondents). The place of origin showed no significant correlation with the answers given (Table 1).

**Table 1:** Characteristics of the study sample.

Parameters	Number of Respondents [n]	Percentage [%]
Gender		
Female	461	56.9
Male	344	42.5
Refusal response	5	0.6
Degree		
Bachelor’s degree/Engineer	732	90.4
Master’s degree	73	9.0
Unified master studies	5	0.6
Voivodship of origin		
Pomorskie	479	59.1
Małopolskie	93	11.2
Warmińsko-mazurske	75	9.3
Kujawsko-pomorskie	37	4.6
Podlaskie	32	4.0
Mazowieckie	19	2.3
Zachodniopomorskie	16	2.0
Lubelskie	15	1.9
Podkarpackie	13	1.6
Śląskie	10	1.2
Łódzkie	8	1.0
Wielkopolskie	7	0.9
Świętokrzyskie	6	0.7
Dolnośląskie	2	0.2
Study profile		
Engineering sciences	499	61.6
Social sciences	202	24.9
Medical and health sciences	52	6.4
Natural sciences	33	4.1
Humanities	22	2.7
Theological sciences	2	0.2
BMI [kg·m^-2^]		
<18.5	52	6.4
18.5–24.9	600	74.1
25.0–29.9	131	16.2
30.0–34.9	23	2.8
35.0–39.9	4	0.5

Participants in the survey reported studying in one of the following fields: engineering sciences– 499 respondents (representing 61.6% of all respondents), social sciences– 202 respondents (24.9% of all respondents), medicine and health sciences– 52 respondents (6.4% of all respondents), natural sciences– 33 respondents (4.1% of all respondents), humanities– 22 respondents (2.7% of all respondents), and theological sciences– 2 respondents (0.2% of all respondents). The field of study did not significantly correlate with the answers given.

### 2.2. Questionnaire and data analysis

This empirical study was conducted using a custom-designed questionnaire with an indirect interview technique via a web-based platform (CAWI) between February 01 and March 31, 2023. Respondents gave informed and voluntary consent to participate in the study, and confirmed that they were familiar with the risk factors associated with taking part in a CAWI survey. The questionnaire of the survey consisted of thematic blocks including: profiling information, respondents’ opinions on individual preferences for physical attractiveness, respondents’ perceptions of physically attractive people, respondents’ potential fears with regard to changing their own appearance, and respondents’ willingness to undertake individual actions in order to obtain a body shape perceived as ideal.

To structure the answers given, respondents answered: "yes" or "no" to each given physical characteristics defined as attractive, their perception of people with an attractive appearance, and their tendency to take individual steps to achieve an ideal physique. The respondents’ concerns regarding potential changes in appearance (weight gain, weight loss, or loss of self-perceived attractiveness) were reported on a 5-point Likert scale (1 –no, 2 –not really, 3 –neither yes nor no, 4 –somewhat, 5 –yes).

The degree of satisfaction with one’s body was calculated from the scores given by the respondents for each body image criterion on a scale of 1 to 5, with "very low" being the lowest (1) and "very high" being the highest (5). Based on the answers given, the sums of the scores obtained for all the features analyzed were calculated for each participant. The higher the number of points obtained, the higher the respondent’s degree of satisfaction with their body. Satisfaction was reported for the following specific aspects of appearance: body weight, height, waist circumference, hip circumference, shoulder width (men)/shoulders (women), chest (men)/breasts (women), abdomen, face.

Total scores (sums of the points) were divided into 3 levels of body satisfaction, with thresholds at 1/3 and 2/3 of the total score range (1-to-5 point scale): low satisfaction level (<1/3 of the range: 1-to-2.33 points), ambivalent satisfaction level (1/3 to 2/3 of the range: 2.34-to-3.66 points), high satisfaction level (> 2/3 range: 3.67-to-5 points). The resulting groups were cross-referenced with the subsequent questions on the form in order to determine the relationships pertaining to the research objective.

Body Mass Index (BMI) was calculated on the basis of the respondents’ anthropometric indices (height and weight). The BMI was used to determine the percentage of respondents with normal body weight, underweight, overweight or obesity. Inference of the results was based on the adopted ranges of BMI, dividing the respondents into four groups: group 1 –respondents with features of underweight or malnutrition (BMI ≤ 18.49 kg·m^-2^), group 2 –respondents with normal body weight (BMI from 18.50 to 24.90 kg·m^-2^), group 3 –respondents with features of overweight (BMI from 25.00 to 29.99 kg·m^-2^), group 4—respondents with features of obesity (BMI ≥ 30.00 kg·m^-2^).

The analysis of the results was carried out taking into account the qualitative variables: gender, BMI, and the body satisfaction score. The results of the study were presented using the percentage distribution (percentage) of individual scores (% of indications). Significance of differences was tested using the Chi-square test with Yates correction. Arithmetic means were calculated for questions on respondents’ concerns about their appearance. Using the Mann-Whitney U-test (men and women were compared) and the Kruskal-Wallis test (groups divided by BMI and satisfaction level with body parts were compared). For all analyses, significance was established at *p* < 0.05. Excel 2000 and Statistica 13.3 (Tibco Software, Palo Alto, USA) were used for the calculations.

## 3. Results

Based on the height and weight given by the respondents, their BMI was calculated and used to classify them into four categories: those with low BMI, those with normal BMI and those with excessive BMI (overweight or obese). The resulting groups were cross-referenced with their answers to the subsequent questions on the questionnaire in order to determine their impact on the answers given. At the outset, respondents were asked to provide their opinion on their individual preference for ‘ideal’ appearance (Table 2). To structure the answers given, the respondents answered: "yes" or "no" for each given characteristic of the individual appearance. The answers given were related to the set of features determining one’s own attractiveness, as presented by the respondents (BMI and self-assessment index, the latter of which was based on the scores for particular criteria of own appearance). Results showed significant differences in the answers given depending on the above-mentioned personal characteristics. The number of responses naming slight underweight as an attribute of attractiveness did not differ along gender lines, whereas the respondents with a normal BMI (77.2%) and those characterised by obesity (76.9%) were significantly (*p*<0.05) less likely to indicate slight underweight as an attribute of an attractive person. Similarly, significant differences were found in the perception of BMI according to one’s satisfaction with body parts. Respondents with low level of satisfaction with body parts were significantly more likely (38.7%) to indicate slight underweight as an attribute of attractive appearance than those with an ambivalent level of satisfaction (25.7%) or a high level of satisfaction with body parts (21.4%). Normal body weight was most frequently indicated as an attribute of attractiveness, regardless of gender, BMI, or satisfaction level with body parts of the participants. Overweight was significantly (*p*<0.05) more often reported as attractive by women (17.6% women) than men (8.4% men). With regards to BMI, respondents characterized by being overweight (26.7%) and obese (26.9%) were significantly (*p*<0.05) more likely to indicate this attribute as indicative of an attractive appearance, in comparison with those with a normal BMI (10.9% of the group) or those who were underweight (5.8% of the group). More women (5.4% of all respondents) reported obesity as a positive image trait than men (0.3% of all respondents). In assessing this attribute, the respondents’ level of satisfaction with body parts also differentiated the study population in a statistically significant way. Those with a low level of satisfaction were significantly (*p*<0.05) more likely (8.0% of the group) to indicate obesity as an attribute of attractiveness than those with an ambivalent level of satisfaction (3.1% of the group), or those with a high level of satisfaction (2.2% of the group) (Table 2).

**Table 2:** Attributes of attractive appearance.

	Gender			BMI					Satisfaction level with body parts			
	Men [%]	Women [%]	*p* *	Underweight and malnutrition [%]	The correct body weight [%]	Overweight [%]	Obesity [%]	*p*	Low [%]	Ambivalent [%]	High [%]	*p*
Severe underweight												
Yes	3.2	5.0	0.21	5.8	4.2	3.8	3.9	0.95	9.3	4.3	2.6	0.36
No	96.8	95.0		94.2	95.8	96.2	96.1		90.7	95.7	97.4	
Slight underweight												
Yes	25.0	25.8	0.79	32.7	22.8	35.1	23.1	**0.02**	38.7	25.7	21.4	**0.01**
No	75.0	74.2		67.3	77.2	64.9	76.9		61.3	74.3	78.6	
The correct body weight												
Yes	89.2	91.5	0.27	86.5	90.1	93.9	92.3	0.38	84.0	91.5	90.8	0.12
No	10.8	8.5		13.5	9.9	6.1	7.7		16.0	8.5	9.2	
Overweight												
Yes	8.4	17.6	**0.00**	5.8	10.9	26.7	26.9	**<0.01**	26.7	13.5	10.3	**0.00**
No	91.6	82.4		94.2	89.1	73.3	73.1		73.3	86.5	89.7	
Obesity												
Yes	0.3	5.4	**0.00**	3.9	2.9	3.8	7.7	0.64	8.0	3.1	2.2	**0.04**
No	99.7	94.6		96.1	97.1	96.2	92.3		92.0	96.9	97.8	
Low height												
Yes	25.9	30.2	0.18	36.5	25.5	38.9	23.1	**0.01**	37.3	26.8	28.4	0.17
No	74.1	69.8		63.5	74.5	61.1	76.9		62.7	73.2	71.6	
Average height												
Yes	75.3	79.8	0.13	75.0	77.32	81.7	80.8	0.64	73.3	80.2	75.3	0.19
No	24.7	20.2		25.0	22.8	18.3	19.2		26.7	19.8	24.7	
High height												
Yes	45.9	53.8	**0.03**	48.1	49.2	55.7	57.7	0.47	68.0	46.2	52.8	**0.00**
No	54.1	46.2		51.9	50.8	44.3	42.3		32.0	53.8	47.2	
Narrow shoulders/arms												
Yes	29.4	36.4	**0.04**	34.6	31.7	42.0	26.9	0.14	49.3	32.0	31.4	**0.01**
No	70.6	63.6		65.4	68.3	58.0	73.1		50.7	68.0	68.6	
Medium shoulders/arms												
Yes	68.3	74.8	**0.04**	80.8	70.3	76.3	73.1	0.23	72.0	70.6	74.5	0.52
No	31.7	25.2		19.2	29.7	23.4	26.9		28.0	29.4	25.5	
Broad shoulders/arms												
Yes	38.1	41.9	0.28	28.9	38.9	48.9	50.0	**0.04**	49.3	38.6	40.6	0.21
No	61.9	58.1		71.1	61.1	51.1	50.0		50.7	61.4	59.4	
Narrow/ slim hips												
Yes	27.9	36.4	**0.01**	30.8	31.4	39.7	34.6	0.33	46.7	30.9	32.1	**0.03**
No	72.1	63.6		69.2	68.6	60.3	65.4		53.3	69.1	67.9	
Medium hips												
Yes	83.1	81.8	0.46	84.6	80.5	86.3	88.5	0.30	80.0	83.2	80.4	0.57
No	16.9	18.9		15.4	19.5	13.7	11.5		20.0	16.8	19.6	
Large/Wide hips												
Yes	30.5	36.4	0.08	30.8	32.7	39.7	38.5	0.43	41.3	34.6	35.8	0.18
No	69.5	63.6		69.2	67.2	60.3	61.5		58.7	68.4	64.2	

*Chi2

Another feature analyzed in terms of its attractiveness was height. Our results indicated that BMI was the only personal characteristic that significantly affected responses regarding short stature. Respondents with a normal BMI were significantly (*p*<0.05) less likely (25.5% of the group) to indicate low height as an attribute of attractiveness when compared to those who were underweight (36.5% of the group) or overweight (38.9% of the group). Average height was the attribute most frequently cited as attractive, regardless of the respondent’s gender, BMI, or satisfaction level with body parts. High height as an attribute of attractiveness was indicated significantly (*p*<0.05) more often by women (53.8% of the group) than men (45.9% of the group) as well as by those with low satisfaction level with body parts (68% of the group) than those with ambivalent satisfaction level (46.2% of the group) or high satisfaction level with body parts (52.8% of the group) (Table 2). The next attribute considered as a potentially attractive was shoulder width. Analysis of the results showed that narrow shoulders were reported to be attractive significantly (*p*<0.05) more often by women (36.4% of the group) than men (29.4% of the group), as well as by respondents with low satisfaction level with body parts (49.3% of the group) more often than by those with ambivalent satisfaction level (32.0% of the group) or those with high satisfaction level with body parts (31.4% of the group). Average shoulder width was the most popular choice as a feature of an attractive appearance across all of the groups discussed. Significant differences in the perception of broad shoulders as an attribute of attractive appearance were only found with regards to BMI. This attribute was indicated more frequently by the overweight and obese individuals (48.9 and 50.0% respectively) than by the normal-weight (38.9% of the group) or the underweight individuals (28.9% of the group) (Table 2).

The last attribute analyzed in terms of its attractiveness was hip width. Narrow hips were reported as attractive significantly more often (*p*<0.05) by women (36.4%) than men (27.9%), as well as by respondents with low satisfaction level with body parts (46.7%) more often than by those with ambivalent satisfaction level (30.9%) or with high satisfaction level with body parts ones (32.1%). BMI did not affect the responses to this question. Average hip width received the most indications as an attribute of attractive appearance regardless of the personal characteristics analyzed (gender, weight, or satisfaction level with body parts of respondents) (Table 2).

Respondents’ self-reported perceptions of attractive people were also defined in terms of selected physical characteristics and eating/health habits (Table 3). For the vast majority of respondents, a person with an attractive appearance is: happy (79.3% of all respondents), healthy (83.8% of all respondents), eating regularly (62.9% of all respondents), exercising (69.4% of all respondents), and slim (56.6% of all respondents). Nevertheless, women, when compared to men, were significantly more likely (*p*<0.05) to select the following characteristics: happy (82.4%), exercising regularly (74.6%), eating regularly (73.1%), and slim (60.3%). The variable ‘BMI’ and ‘self-reported satisfaction with body image’ had no significant effect (*p*>0.05) on how the respondents viewed these characteristics. Furthermore, about half of the respondents–regardless of gender, BMI, or satisfaction level with body parts–rated an attractive person as "better perceived by others". Least important to respondents in assessing their own body were features related to physical activity: strongly muscular (29.05% of all respondents), athletic body build (27.1% of all respondents), engagement in competitive sports (32.4% of all respondents), or sports as a hobby (34.3% of all respondents). Respondents with low satisfaction with their body parts (40.0%) were significantly more likely (*p*<0.05) to perceive an attractive person as being strongly muscular, compared to those expressing neutral attitudes (25.9%), or those highly satisfied with their body appearance (31.7%). In contrast, ‘athletic physique’ and ‘playing sports as a hobby’ were more significantly frequent (*p*<0.05) answers for women and those unsatisfied with their body image. On the other hand, gender, BMI, or satisfaction level with body parts did not affect (*p*>0.05) responses for the attribute “playing sport competitively”. For a significant minority of the respondents, the image of a person with an attractive appearance was not associated with dieting (24.9% of total respondents), or slimming down (14.4%of all respondents). It was noted, however, that for those with lower levels of satisfaction with their body parts, going on a diet (42.7%), or losing weight (26.7%) were more important (*p*>0.05) in assessing an attractive appearance. The study also showed that for the vast majority of young respondents, a person with an attractive appearance is not a female influencer (78.9% of all respondents) and not a male influencer (81.1% of all respondents). But, it was noted that gender and level of satisfaction with body parts influenced the distribution of ratings of these variables between groups (*p*>0.05). For men and those with high satisfaction level with body parts, the notion of an attractive person was significantly more likely to be associated with a non-influencer/influencer. For respondents, the image of a person with an attractive appearance is definitely not associated with: obesity (93.9% of all respondents), smoking (93.1% of all respondents), or starvation (95.9% of respondents). It is noteworthy, however, that the group of respondents who named obesity and smoking of tobacco products as attractive were predominantly women (8.9% and 8.9%, respectively), or those with a low level of satisfaction with their body image (14.7% and 18.7%, respectively) (Table 3).

**Table 3:** Respondents’ perceptions of a person with an attractive appearance.

	Gender			BMI					Satisfaction level with body parts			
	Men [%]	Women [%]	*p* *	Underweight and malnutrition [%]	The correct body weight [%]	Overweight [%]	Obesity [%]	*p*	Low [%]	Ambivalent [%]	High [%]	*p*
Happy												
Yes	76.2	82.4	**0.03**	90.4	79.7	74.8	84.6	0.09	77.3	80.6	21.0	0.75
No	23.8	17.6		9.6	20.3	25.2	15.4		22.7	19.4	78.0	
Healthy												
Yes	83.4	84.2	0.78	88.5	84.7	77.1	88.5	0.12	78.7	83.4	86.0	0.29
No	16.6	15.8		11.5	15.3	22.9	11.5		21.3	16.6	14.0	
Slim												
Yes	52.9	60.3	**0.04**	59.6	58.1	53.4	50.0	0.66	66.7	57.1	54.6	0.17
No	47.1	39.7		40.4	41.9	46.6	50.0		33.3	42.9	45.4	
Obese												
Yes	3.2	8.9	**0.00**	5.8	6.5	6.9	3.8	0.93	14.7	6.3	4.4	**0.01**
No	96.8	91.1		94.2	93.5	93.1	96.2		85.3	93.7	95.6	
Better seen by others												
Yes	52.3	56.8	0.20	46.2	55.2	54.2	69.2	0.28	61.3	55.8	51.7	0.28
No	47.7	43.2		53.8	44.8	45.8	30.8		38.7	44.2	48.3	
Strongly muscular												
Yes	27.9	30.2	0.49	25.0	30.7	25.9	19.2	0.37	40.0	25.9	31.7	**0.02**
No	72.1	69.8		75.0	69.3	74.1	80.7		60.0	74.1	68.3	
Using diets												
Yes	19.8	29.9	**0.00**	28.9	24.3	29.0	30.8	0.59	42.7	23.3	24.7	**0.00**
No	80.2	70.1		71.1	75.7	71.0	69.2		57.3	76.7	75.3	
Slimming down												
Yes	12.2	16.5	0.09	13.5	13.8	19.1	15.4	0.50	26.7	13.7	12.9	**0.01**
No	87.8	83.5		86.5	86.2	80.9	84.6		73.3	86.3	87.1	
Exercising regularly												
Yes	64.2	74.6	**0.00**	75.0	71.6	61.8	69.2	0.15	72.0	70.8	68.6	0.77
No	35.8	25.4		25.0	28.4	38.2	30.8		28.0	29.2	31.4	
Smoking												
Yes	4.9	8.9	**0.03**	11.5	6.9	6.1	11.5	0.52	18.7	6.3	5.5	**0.00**
No	95.1	91.1		88.5	93.1	93.9	88.5		81.3	93.7	94.5	
Eating regularly												
Yes	52.6	73.1	**0.00**	75.0	64.6	58.0	69.2	0.15	70.7	65.1	61.3	0.28
No	47.4	26.9		25.0	35.4	42.0	30.8		29.3	34.2	38.7	
Using a hunger strike												
Yes	2.9	5.4	0.08	5.8	3.9	6.1	3.9	0.69	10.7	3.3	4.4	0.10
No	97.1	94.6		94.2	96.1	93.9	96.1		89.3	96.7	95.6	
Model/influencer (female)												
Yes	16.6	25.6	**0.00**	25.0	21.0	24.4	19.2	0.76	34.7	15.8	23.6	**0.00**
No	83.4	74.4		75.0	79.0	75.6	80.8		65.3	81.5	76.4	
Model/influencer (male)												
Yes	13.7	24.1	**0.00**	21.2	19.1	22.1	15.4	0.80	32.0	16.6	21.4	**0.01**
No	86.3	75.9		78.8	80.1	77.9	84.6		68.0	83.4	78.6	
Engaged in competitive sport												
Yes	29.4	35.4	0.07	34.6	33.6	32.1	15.4	0.23	34.7	32.7	32.5	0.93
No	70.6	64.6		65.4	66.4	67.9	84.6		65.3	67.3	67.5	
Practising sport as a hobby												
Yes	27.0	41.6	**0.00**	36.5	33.7	40.5	46.2	0.32	53.3	32.0	36.1	**0.00**
No	73.0	58.4		63.5	66.3	59.9	53.8		46.7	68.0	63.9	
Athletic physique												
Yes	22.7	31.5	**0.01**	28.9	27.2	30.5	23.1	0.82	37.3	24.4	30.6	**0.03**
No	77.3	68.5		71.2	72.8	69.5	76.9		62.7	75.6	69.4	

*Chi2

Respondents’ expressed concerns about their appearance were rated on a 5-point Likert scale, according to the qualitative variables studied (gender, BMI and satisfaction level with their body parts) (Table 4). Just over half of the total respondents (55.5%) declared their concerns about gaining weight. Gender, BMI, or satisfaction level with one’s body image had significant effects (*p*<0.05) on the response distributions. Concerns about gaining weight were expressed significantly more often (*p*<0.05) by: women (‘somewhat’ and ‘yes’ 69.0%, mean scores of 3.87), obese respondents (73.1%, mean scores of 4.04), or those with a lower degree of satisfaction with their body (82.7%, mean scores of 4.39). In contrast, 12.6% of all respondents expressed their concerns about weight loss, with significant differences found according to gender. Men (16.9%, mean scores of 1.94) compared to women (8.3%, mean scores of 1.58) were significantly more likely to indicate that they were concerned about weight loss. In contrast, 30.5% of all respondents answered ‘yes’ and ‘somewhat’ to the question "Do you fear that your appearance will change to the point that you will no longer be an attractive person". Women (40.6%, mean scores of 2.78) and those with low satisfaction level with their body parts (54.6%, mean scores of 2.44) expressed statistically higher concerns (*p*<0.05) about an adverse change in appearance (Table 4).

**Table 4:** Respondents’ concerns about their appearance.

	Gender			BMI					Satisfaction level with body parts			
	Men [%]	Women [%]	*p*	Underweight and malnutrition [%]	The correct body weight [%]	Overweight [%]	Obesity [%]	*p*	Low [%]	Ambivalent [%]	High [%]	*p*
Gaining weight												
not	43.9	16.5	**<0.01** ^**a**^	46.2	28.4	23.7	11.5	**<0.01** ^**a**^	8.0	22.5	43.5	**0.00** ^**a**^
rather not	7.3	5.4		13.5	5.9	3.8	11.5		1.3	5.4	8.8	
neither yes nor no	6.7	9.1		3.8	9.6	3.8	3.9		8.0	7.0	10.0	
rather yes	5.2	12.8		7.7	8.8	13.8	7.7		9.3	10.2	8.5	
yes	36.9	56.2		28.8	47.3	54.9	65.4		73.4	54.9	29.2	
mean ^b^	2.84	3.87	**<0.01** ^**c**^	2.60	3.41	3.73	4.04	**<0.01** ^**d**^	4.39	3.70	2.71	**<0.01** ^**d**^
Weight Loss												
not	62.8	72.7	**<0.01** ^**a**^	55.8	67.8	75.6	73.1	0.08^a^	74.7	68.6	66.4	0.51^a^
rather not	10.5	10.8		9.6	10.8	9.9	11.5		8	11.1	10.7	
neither yes nor no	9.9	8.2		7.7	9.4	7.6	7.6		9.3	9.2	8.5	
rather yes	3.5	2.2		7.7	2.9	0.0	3.9		4	2.8	2.2	
yes	13.4	6.1		19.2	9.1	6.9	3.9		4	8.3	12.2	
mean ^b^	1.94	1.58	**<0.01** ^**c**^	2.25	1.74	1.53	1.54	**0.02** ^**d**^	1.55	1.71	1.83	0.31^d^
Your appearance will change so much that you will no longer be an attractive person												
not	61.9	43.6	**<0.01** ^**a**^	51.9	51.2	51.2	57.6	0.97^a^	28.1	50.3	59.8	**0.00** ^**a**^
rather not	8.4	5.9		7.7	6.8	6.9	7.7		4	6.3	8.9	
neither yes nor no	9.3	10.0		9.6	10.1	9.2	3.9		13.3	8.5	10.7	
rather yes	3.8	10.4		7.7	7.2	7.6	15.4		5.3	10.0	4.1	
yes	16.6	30.2		23.1	24.7	25.1	15.4		49.3	24.9	16.5	
mean ^b^	2.05	2.78	**<0.01** ^**c**^	2.42	2.47	2.49	2.23	0.89^d^	3.44	2.53	2.09	**<0.01** ^**d**^

^a^ Chi2

^b^ Likert scale

^c^ U-Manna Whitneya

^d^ Kruskala-Wallisa

Respondents’ self-reported propensity for specific behaviors to achieve their dream body shape/appearance was also assessed (Table 5). Almost 21% of all respondents declared full satisfaction with the appearance of their own body, with significantly more often (*p*<0.05) expressed by: men (25.0%), those with normal BMI (22.7%), or those with ambivalent satisfaction level with body parts (38.0%). Vast majority of respondents (78.8% of the total) were willing to exercise more in order to achieve the ideal figure. This attitude was expressed significantly more often (*p*<0.05) by women (82.0%) or by the respondents with low satisfaction with their body image (85.3%). In addition, women or those unsatisfied with their body image were significantly more likely (*p*<0.05) to: eat healthier (75.7% and 81.3%, respectively), pay attention to ingredients in their food (62.3% and 70.7%, respectively) or the calorie counts (49.7% and 65.3%, respectively), choose clothes that disguise their body shape (33.8% and 58.7%, respectively), or work with a personal trainer (45.6% and 56.0%, respectively) (Table 5). On the other hand, dieting to achieve an ideal appearance was declared by 46.9% of all respondents. These were significantly more likely to be women (50.8%), those with a overweight (55.7%), or those with a high level of satisfaction with their body image (49.2%). The present study also assessed the willingness of respondents to compose their own meals and to take advice from a dietician. Significant differences (*p*<0.05) were found in the distribution of scores for these variables, depending on gender, BMI, or satisfaction level with one’s own body parts. Women were significantly more likely (*p*<0.05) than men to declare their willingness to compose their own meals (54.2% vs. 39.0%, respectively) and to take advice from a dietician (42.7% vs. 33.4%, respectively). In addition, overweight and obese persons (57.3% and 57.7% respectively) or those with low satisfaction level with body parts (62.7%) were also more inclined to compose their own meal (Table 5).

**Table 5:** Respondents’ stated propensity to engage in selected behaviours to achieve their dream physique/appearance.

	Gender			BMI					Satisfaction level with body parts			
	Men [%]	Women [%]	*p* *	Underweight and malnutrition [%]	The correct body weight [%]	Overweight [%]	Obesity [%]	*p*	Low [%]	Ambivalent [%]	High [%]	*p*
full satisfaction with the appearance of your own body												
yes	25	16.9	**0.00**	15.4	22.7	14.5	7.7	**0.03**	8.0	38.0	12.0	**0.00**
no	75	83.1		84.6	77.3	85.5	92.3		92.0	62.0	88.0	
exercise more												
yes	75.6	82.0	**0.02**	80.8	79.2	77.1	88.5	0.64	85.3	70.1	83.7	**0.00**
no	24.4	18.0		19.2	20.8	22.9	11.5		14.7	29.9	16.3	
use diets												
yes	43.9	50.8	0.05	42.3	45.5	55.7	42.3	**0.01**	24.0	37.6	49.2	**0.00**
no	56.1	49.2		57.7	54.5	44.3	57.7		76.0	62.4	50.8	
pay attention to ingredients in their food												
yes	45.1	62.3	**0.00**	53.9	52.9	61.8	69.2	0.12	70.7	50.2	55.1	**0.01**
no	54.9	37.7		46.1	47.1	38.2	30.8		29.3	49.8	44.9	
eat healthier												
yes	57.8	75.7	**0.00**	61.6	67.6	70.2	80.8	0.34	81.3	59.0	71.2	**0.00**
no	42.2	24.3		38.4	32.4	29.8	19.2		18.7	41.0	28.8	
compose your own meals												
yes	39.0	54.2	**0.00**	38.5	46.0	57.3	57.7	**0.04**	62.7	43.2	47.9	**0.01**
no	61.0	45.8		61.5	54.0	42.7	42.3		37.3	56.8	52.1	
pay attention to the calorie counts												
yes	36.0	49.7	**0.00**	34.6	42.6	48.9	65.4	**0.04**	65.3	43.5	40.5	**0.00**
no	64.0	50.3		65.4	57.4	51.1	34.6		34.7	56.5	59.5	
take advice from a dietician												
yes	33.4	42.7	**0.01**	26.9	37.8	47.3	42.3	0.06	56.0	32.8	39.4	**0.00**
no	66.6	57.3		73.1	62.2	52.7	57.7		44.0	67.2	60.6	
choose clothes that disguise their body shape												
yes	14.0	33.8	**0.00**	17.3	25.2	29.0	26.9	0.41	58.7	18.5	24.0	**0.00**
no	86.0	66.2		82.7	74.8	71.0	73.1		41.3	81.5	76.0	
work with a personal trainer												
yes	32.0	45.6	**0.00**	40.4	39.9	37.4	46.2	0.86	56.0	35.1	39.9	**0.00**
no	68.0	54.4		59.6	60.1	62.6	53.8		44.0	64.9	60.1	

*Chi2

## 4. Discussion

The present study aimed to identify declared patterns of ideal appearance among young adults, specifically students at selected universities in Poland. Based on the BMI calculations performed in the present study, most of the respondents had a normal BMI values. These individuals perceived normal body mass as attractive. Furthermore the respondents tended to indicate the normal body weight as an attribute of attractiveness, regardless of their gender, BMI or satisfaction with body parts. The respondents with lower body weight, categorized as slightly underweight, were less likely to be perceived as attractive by students. In an earlier study, scholars asked students about their impressions about gender attractiveness, and noted that they considered people with normal BMI values as attractive [51]. This may, therefore, indicate the congruence with our findings. The results of our study related to BMI show that overweight respondents were significantly more likely to indicate this feature as indicative of an attractive appearance compared to those with normal BMI or underweight. Increased body weight was cited as a positive image trait significantly more often (*p<0*.05) by female respondents. Undoubtedly, however, basing body attractiveness solely on Body Mass Index (BMI) as a measure of body proportionality is not authoritative since it is not an adequate predictor of individual health status [52]. BMI is currently a screening tool for assessing the reference point in obesity classification but, like all anthropometric measurements, it is only a surrogate measure of body fatness [53]. People with a similar BMI may have different body shapes in terms of fat tissue and muscle mass [54, 55]. Similar observations have been made by other authors. For instance, Priya et al. [56] reported that women (college students) of which 77.5% had normal BMI perceived their body image as correct, whereas women with a lower body weight (underweight) took steps to increase their body weight. Similar observations have been made by other authors. For instance Pirya et al. [56], reported that women (college students) of which 77.5% had normal BMI perceived their body image as correct, whereas women with a lower body weight (underweight) took steps to increase their body weight.

Recent data on the contemporary human population showed that height is positively correlated with indicators of social status, including: occupational attainment or education and income, which held true for both males and females. Height can also influence how people perceive themselves [57]. In our study, average height was the height frequently indicated as attractive, regardless of gender or BMI.

Furthermore, our results show that narrow shoulders and narrow hips were more frequently indicated by women than men as an attribute of attractiveness. These findings are not supported by the available literature on the perceived attractiveness of women’s hip and shoulder width [57, 58]. There is a widespread perception that very low waist-to-hip ratios and low body mass indexes (BMIs) in women are judged as attractive by men in well-nourished populations, as these features may indicate, for example, higher fertility [59]. In our study, average hip width was the one most often reported as attractive, regardless of the respondent profile. The distribution of responses did not depend on the gender, weight or height of the respondents. A previous study [60] on the assessment of waist-to-hip ratio attractiveness in women showed pronounced inter-individual differences across the sample. WHR values from low to medium (i.e., 0.65-to-0.75) were usually considered the most attractive. Values above the mean (>0.75) or values below the normal range of the trait (<0.65) were less frequently preferred. The analysis of the literature data [60, 61] indicates that the lower the WHR, the more attractive the female figure was. In another study, a wide upper body and narrow waist (classic V-shape) was demonstrated as a major predictor of male attractiveness for the women respondents [58]. Pawłowski [61] indicates that other factors influencing women’s perception of men’s attractiveness include body height but also the relative length of legs, i.e., overall body length proportions. In turn, men prefer longer legs in women.

Physical self-esteem is an important factor influencing young people’s well-being. This factor is particularly relevant during adolescence and intensive development, as this is the period when personality is formed and is closely linked to young people’s mental health and well-being [62]. Body image is an important part of the complex mechanism that is self-identity. The socio-cultural environment can cause different body image distortions and subjective disorders [63]. Our study has shown that person with an attractive physical appearance is a person: happy, healthy, eating regularly, undertakes regular physical activity. In recent decades, interest in the male figure and bodybuilding has increased substantially in the societies of the developed countries [64]. This trends poses a risk of developing a psychological disorder involving a subjective belief in an unsightly appearance or physique–muscle dysmorphia (MD) [65]. Unlike *anorexia nervosa* (AN, eating disorder), the main focus and concern in MD is inadequate body musculature or preoccupation with other aspects of appearance, as in the body dysmorphic disorder (BDD) [66–68]. Our study demonstrated that women place a higher value on maintaining an attractive appearance through dieting than men. Importantly, neither women nor men respondents identified the influencers as role models of attractive appearance. Research conducted among young adults has shown that for approximately 30–40% of them, social media was a space that significantly influenced attitudes towards their own bodies. The media are a place for creating one’s own image, a source of motivation to engage in behaviors aimed at improving the appearance of the body and taking care of its appearance but also a space for social comparisons relating to the appearance of one’s own body [69]. Self-perception of the body on social media can be seen as a kind of self-advertisement that serves to affirm the users’ attractiveness, as well as an occasion for identifying current trends in this respect [70]. Nearly half of the young women and men surveyed in our study expressed concern over their body satisfaction in the future. Similar observations were made by other authors [71], indicating that social media use and contact, particularly the strongly visual one, and the appearance-focused content were related with poorer perceptions of body image. Women, for whom appearance is strongly associated with attractiveness and self-esteem, may be at increased risk of negative perceptions of body image and the development of nutrition disorders. Based on an ongoing meta-analysis [72], the body ideal promulgated in social media has a moderate negative impact on the audience’s perception of body image. As per the same study, women are more likely to report anxiety about their own appearance and are more likely to define themselves through the lens of appearance putting themselves at risk of a number of mental health issues [72]. This has been confirmed by our findings, wherein women were more likely to voice concerns about becoming less attractive. Other published studies have also shown that women compared themselves with others in terms of their appearance more often than men [69].

We also assessed the willingness of young women and men to engage in selected behaviors to achieve their dream body shape. Excessive attention to food, restrictive diets, perfectionism, co-occurring anxiety, need for control, and rigid food preparation behaviors and rituals may predispose one to develop orthorexia [73]. Athletes [74–76], dietetic students [77, 78], and vegetarians, those who follow a rigid eating plan and spend a lot of time preparing meals, are particularly at risk of developing orthorexia [74, 79]. Studies of the physiotherapy students’ attitudes towards physical activity found that female students were open to the idea about the need to participate in compulsory sports activities [80]. Weight loss practices were undertaken by university students, as per several other studies [81, 82]. Dissatisfaction with body image is widely regarded as an important public health concern [3, 51]. About half of the students reported trying to control their body weight in order to have a real impact on improving their appearance and indirectly influence the feeling of emotion. Some emotions can generate positive actions or reactions, such as joy or satisfaction with one’s body image, but there are other emotions (anger, sadness, aversion) that may cause harmful feelings for the individual [83]. In this case, an appropriate psychological intervention [66] could mediate the impact of both positive and negative emotions on young people’s behavior, including their body image.

## 5. Conclusions

Results of the survey on body image of young adults studying at selected universities in Poland revealed that attitudes towards one’s physical appearance are diverse and also influenced by various factors. Respondents expressed readiness to undertake a range of activities related to increased exercise, changes to eating habits or seeking assistance (e.g., of a personal trainer) to achieve the desired body shape.

Our findings can be used as cornerstones for discussion and consideration towards developing the educational activities on nutrition, as well as on overweight*-* and obesity-related health issues. It is prudent to develop good eating habits and promote daily physical activity as early as in nursery education, so as to promote body satisfaction among young adults. It is also important that young adults are aware of the complexity of factors that influence their self-perception through the lens of observed patterns and forming trends, and that they try to approach it with detachment and self-acceptance.

Our study does have some limitations. Our research was conducted within a narrow sample (university students); therefore the results obtained cannot be generalized to the entire segment of young people in Poland. The scope of the presented study is also limited, as it focuses on a selected component of attitudes towards one’s own body and inclination to change them. The results obtained are, therefore, a starting point for further research. In the future, it would be sound to verify our empirical data on a larger sample of young adults in respect of the typology of this group defined in literature. Further research in this area could be aimed as identifying other variables that were not included in this study, for example environmental factors: eating behavior and habits, physical activity, drugs and caffeine using and eating disorders. Moreover, considering the research tool used, the study could be extended to measure parameters related to the assessment of the attractiveness of individual body parts not only in terms of the opposite sex but also in one’s own sex. The literature works emphasize that, apart from BMI (which does not distinguish between fat and muscle tissue), the measurement of waist circumference is a more reliable indicator for assessing normal body weight, overweight and obesity, whereas the measurement of arm circumference–for assessing undernutrition. Hence, these indicators should be deployed in future studies to identify people with normal or abnormal (undernutrition, overweight and obesity) nutritional status.
